# Comparison of procalcitonin and high-sensitivity C-reactive protein for the diagnosis of sepsis and septic shock in the oldest old patients

**DOI:** 10.1186/s12877-017-0566-5

**Published:** 2017-08-01

**Authors:** Hongmin Zhang, Xiaoting Wang, Qing Zhang, Ying Xia, Dawei Liu

**Affiliations:** Department of Critical Care Medicine, Peking Union Medical College Hospital, Chinese Academy of Medical Sciences, 1# Shuai Fu Yuan, Dong Cheng District, Beijing, 100730 China

**Keywords:** Procalcitonin, High-sensitivity C-reactive protein, Sepsis, Septic shock, Oldest old

## Abstract

**Background:**

Although the role of serum procalcitonin (PCT) and high-sensitivity C-reactive protein (hs-CRP) in the diagnosis of sepsis and septic shock is well studied, it has not been investigated among oldest old patients. The aim of our study is to determine the role of PCT and hs-CRP in the assessment of sepsis and septic shock in this specific group of patients in the ICU.

**Methods:**

This is a prospective observational study. Patients >85 years of age admitted to the ICU from May 1st, 2016 to February 1st, 2017 were evaluated. Patients were divided into a sepsis and septic shock group(sepsis/SS) and a non-sepsis group. Serum levels of PCT, hs-CRP and the WBC were measured within 12 h of admission.

**Results:**

A total of 70 patients aged 85 years and older were enrolled in this study. Fifty patients were labelled as sepsis/SS and the other 20 were labelled non-sepsis. A ROC analysis showed that the area under the curves (AUC) of hs-CRP and PCT for the discrimination of sepsis/SS patients were 0.825 (95% confidence interval[CI]: 0.73-0.92; *P* < 0.001) and 0.819 (95%CI:0.72-0.92; *p* < 0.001), respectively. In a subgroup analysis of the sepsis/SS group, 27 patients had sepsis, while the other 23 patients had septic shock. The ROC analysis showed that the AUCs of hs-CRP and PCT for the discrimination of septic shock patients from sepsis patients were 0.751 (95% CI: 0.62-0.88; *P* = 0.002) and 0.719 (95% CI:0.57-0.86; *p* = 0.007), respectively.

**Conclusion:**

For the oldest old patients, hs-CRP is not inferior to PCT in the diagnosis of sepsis and septic shock.

## Background

According to population statistics, individuals 85 years and older (oldest old) make up 1.8% of the total population but they account for 8% of all hospital discharges [1]. The oldest old age group is the fastest growing segment of the elderly population [2, 3]. As the geriatric population continues to grow, the number of oldest old patients admitted to the intensive care unit (ICU) will increase accordingly.

Sepsis is the leading cause of mortality in critically ill patients. Despite that the overall mortality rate of sepsis patients is declining, the incidence of sepsis and the number of sepsis-related deaths are still increasing [4]. The elderly, especially those older than 80 years of age, frequently display non-specific signs and symptoms of acute infection that often present the physician with a diagnostic challenge [5]. The ability to diagnose or exclude suspected sepsis is vitally important for patient outcomes.

Procalcitonin **(**PCT) and high-sensitivity C-reactive protein (hs-CRP) are the most frequently used biomarkers for critically ill patients in whom sepsis is suspected [6]. PCT is usually considered to have a higher capacity than hs-CRP in the diagnosis of sepsis [7, 8]. However, whether PCT is still more useful than hs-CRP in diagnosing sepsis in oldest old patients has not been investigated. We hereby performed this study to compare the efficacy of PCT and hs-CRP in the diagnosis of sepsis and septic shock in oldest old patients.

## Methods

### Study design and patient population

This is a prospective observational study conducted at Peking Union Medical College Hospital. Patients aged above 85 years and older admitted to intensive care unit from May 1st, 2016 to February 1st, 2017 were studied. Patients were admitted either from medical ward where the patients have been treated for several days to several weeks or from emergency room. The main reasons that they were transferred to ICU include respiratory failure, shock, renal failure and high risk surgery. Based on the evaluation of physician, blood tests were taken when infection was suspected. The diagnosis of sepsis was made based on the new definition developed by the Sepsis Definitions Task Force: sepsis is defined as life-threatening organ dysfunction caused by a dysregulated host response to infection. Organ dysfunction can be identified as an acute change in total SOFA score 2 points subsequent to the infection. Septic shock is defined as sepsis patients who have persistent hypotension that requires vasopressors to maintain a MAP ≥ 65 mmHg and who have a serum lactate level > 2 mmol/L despite adequate volume resuscitation [9]. Patients were excluded if they had any of the following conditions: a lack of informed consent, survival less than 12 h, neutropenia, chemotherapy during the previous 90 days, or the withholding of life support.

### Laboratory testing

All blood samples were drawn within 12 h of ICU admission. The PCT level was measured using the electrochemical luminescence method (VIDAS Brahms PCT, Mannheim, Germany) and the CRP level was measured using an immunoturbidimetric assay (Beckman, Carlsbad, CA 92010, USA). Lactate levels were measured using a blood-gas analyser (GEM 3000, USA).

### Statistical analysis

Statistical analysis was performed using the SPSS version 13.0 statistical software package (SPSS Inc., Chicago Illinois, USA). Continuous data were expressed as the mean ± standard deviation when normally distributed. Non-normally distributed variables were expressed as medians and interquartile ranges. Categorical variables were presented as numbers and percentages. Differences in clinical and laboratory findings were assessed using unpaired t-tests, Mann-Whitney U tests and chi-square tests or exact Fisher’s tests, when appropriate. A correlation analysis between PCT, hs-CRP and SOFA score was performed using a nonparametric Spearman’s test. Receiver operating characteristic (ROC) curves and the areas under each respective curve were calculated. The maximum PCT and CRP concentrations, and the white blood cell (WBC) in the first 12 h were used to calculate the ROC curves. Statistical significance was defined as p<0.05.

### Ethics, consent and permissions

The study was conducted according to the Declaration of Helsinki and was approved by the ethics committee of our institution (Approval Number: JS1300). Written informed consent was obtained from all patients or the next of kin if patients were unconscious or in a state of altered mentation.

## Results

### General characteristics of all patients

A total of 70 patients aged 85 years and older were enrolled in this study. Fifty patients were labelled sepsis/SS, and the other 20 patients were labelled non-sepsis. The general characteristics are illustrated in Table 1. The two groups showed no difference regarding age. The mean ages in the sepsis/SS group and the non-sepsis group were 92.6 years and 92.7 years respectively, and 81.6% vs 75.0% were men, respectively. The sepsis/SS group had a higher peak temperature (38.2 °C vs 37.4 °C, *P* = 0.001) and higher lactate level (2.3 mmol/L vs 1.3 mmol/L, *P* = 0.023). The sepsis/SS group had a higher SOFA sore (8 vs 3, P<0.001). The sepsis/SS group had a higher 28-day mortality than the non-sepsis group, but the difference was not statistically significant (16% vs 5%, *p* = 0.207).

**Table 1:** General characteristics of the sepsis/SS and non-sepsis group

Categories	Sepsis/SS group(*n* = 50)	Non-sepsis group(*n* = 20)	*p* value
Age (yr)	92.6 ± 4.5	92.7 ± 3.2	0.992
Sex (male, %)	40 (81.6%)	15 (75.0%)	0.553
Diagnosis			
Pneumonia	37 (74.0%)	0	-
Biliary tract infection	6 (12.0%)	0	-
Enteral infection	3 (6.0%)	0	-
UTI	3 (6.0%)	0	-
CRBSI	1 (2.0%)	0	
Cerebral disease^a^	0	8 (40.0%)	-
Heart failure	0	5 (25.0%)	-
GIB	0	3 (15.0%)	-
Renal failure	0	2 (10.0%)	-
asthma	0	2 (10.0%)	-
Medical history			
Stroke	27 (54.0%)	11 (55.0%)	0.958
Cancer	16 (32.0%)	6 (30.0%)	0.864
CKD	12 (24.0%)	7 (35.0%)	0.341
CAD	29 (58.0%)	13 (65.0%)	0.589
HTN	36 (72.0%)	15 (75.0%)	0.812
DM	14 (28.0%)	8 (40.0%)	0.333
COPD	13 (26.0%)	5 (25.0%)	0.952
Peak Temperature (°C)	38.2 (37.6-38.7)	37.4 (36.6-37.9)	0.001
Lactate(mmol/L)	2.3(1.4-3.4)	1.3(1.0-1.8)	0.023
SOFA	8(5-10)	3(2-4)	0.000
28 day mortality	8(16.0%)	1(5.0%)	0.207

*UTI* urinary tract infection, *CRBSI* catheter-related bloodstream infection, *GIB* gastrointestinal bleeding, *CKD* chronic kidney dysfunction, *CAD* coronary artery disease, *HTN* hypertension, *DM* diabetes mellitus, *COPD* chronic obstructive pulmonary disease, *SOFA* sequential organ failure assessment

^a^cerebral disease: acute stroke, head injury, status epilepsy

### WBC, PCT and hs-CRP between sepsis/SS and non-sepsis group

No difference was found in the WBC between the two groups. The CRP level and PCT level in the sepsis/SS group was significantly higher than that of the non-sepsis group. (Table 2).

**Table 2:** Biomarkers between sepsis/SS and non-sepsis group

Categories	Sepsis/SS group(*n* = 50)	Non-sepsis group(*n* = 20)	*p* value
WBC (×10^9^/L)	11.9 (7.9-15.3)	9.7 (7.3-13.4)	0.170
hs-CRP (mg/L)	143.4 (82.6-212.4)	50.9 (32.3-74.1)	0.000
PCT (ng/ml)	0.84 (0.36-5.8)	0.08 (0.05-0.54)	0.000

*WBC* white blood cell, *hs-CRP* high-sensitivity C-reactive protein, *PCT* procalcitonin

The hs-CRP level and SOFA score were well correlated (*R* = 0.686, P<0.001). The PCT level and SOFA were also correlated (*R* = 0.641, P<0.001). The WBC level and SOFA score were poorly correlated (*R* = 0.037, *P* = 0.764).

**Fig. 1:**
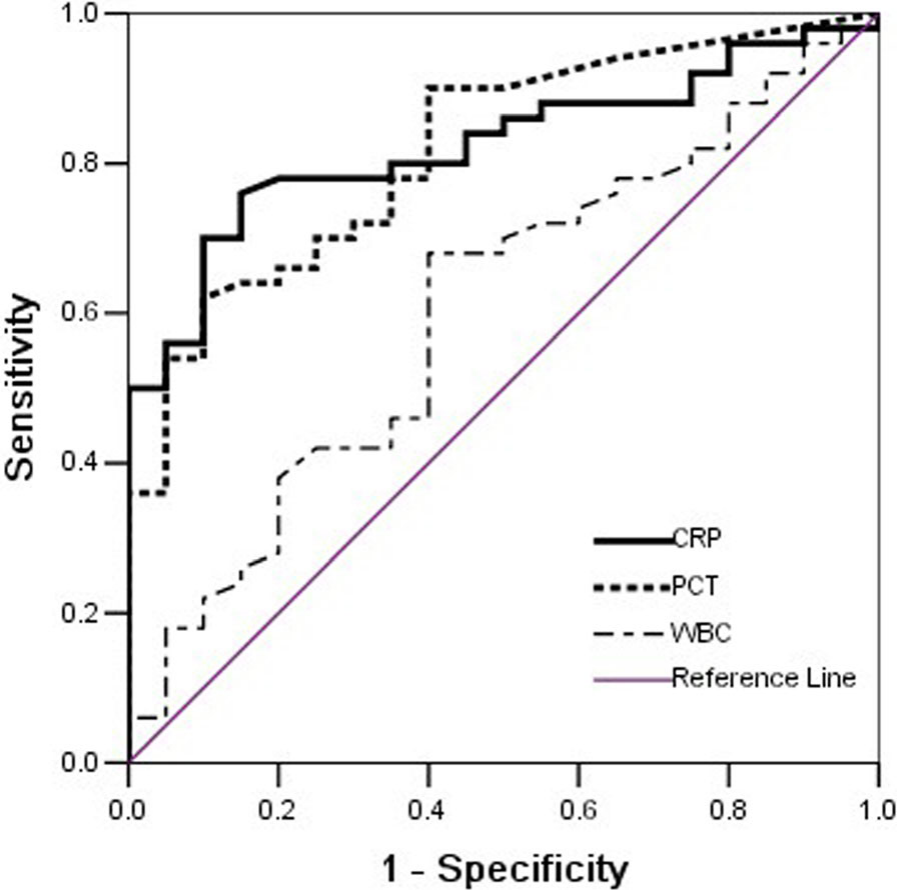
Diagnostic values of CRP, PCT, WBC for sepsis/SS, estimated by receiver operating curve (ROC) analysis. CRP area 0.825 (95% CI 0.73-0.92), *P <* 0.001; PCT area 0.819 (95% CI 0.71-0.92), *P* < 0.001; WBC area 0.606 (95% CI 0.46-0.75), *P* = 0.170. sepsis/SS: sepsis/septic shock

To evaluate the sensitivity and specificity of these three biomarkers, ROC curves were calculated (Fig. 1). The ROC analysis showed that hs-CRP was a good marker for the discrimination of sepsis/SS patients, with an area under the curve (AUC) of 0.825 (95% confidence interval[CI]: 0.73-0.92; P<0.001). PCT was found to have an AUC of 0.819 (95%CI: 0.72-0.92; p<0.001), which was not statistically significant compared to hs-CRP (Z = 0.084, *P* = 0.933). The WBC was found to have an AUC of 0.606 (95% CI:0.46-0.75; *p* = 0.170).

For serum hs-CRP, the optimum cut-off value was 74.2 mg/L, which resulted in a sensitivity of 78.0%, a specificity of 75.0%, a positive predictive value (PPV) of 88.6% and a negative predictive value (NPV) of 57.7%. For serum PCT, the optimum cut-off value was 0.45 ng/ml, which resulted in a sensitivity of 72.0%, a specificity of 70.0%, a PPV of 85.7% and an NPV of 50.1%. For a serum PCT value of 0.25 ng/ml, the sensitivity and specificity were 88.0% and 65.0%, PPV and NPV were 86.2% and 68.4%, respectively.

### Subgroup analysis of sepsis/SS group

Of the 50 sepsis/SS patients, 27 patients had sepsis, while the other 23 patients had septic shock. The two groups showed no differences in either age or sex. The sepsis group had more pneumonia patients, but this result was not statistically significant (23/27 vs 14/23, *p* = 0.052). The sepsis group had less patients with biliary infections (1/27 vs 5/23, *p* = 0.048). The sepsis and septic shock group had similar peak temperatures (38.0 °C vs 38.3 °C, *p* = 0.283). The septic shock group had higher lactate levels (2.3 mmol/L vs 1.3 mmol/L, p<0.001) and higher SOFA scores (10 vs 6, p<0.001). No difference was found regarding comorbidities between the two groups. The septic shock group had a higher mortality rate (30.4% vs 3.7%, *p* = 0.011).

**Fig. 2:**
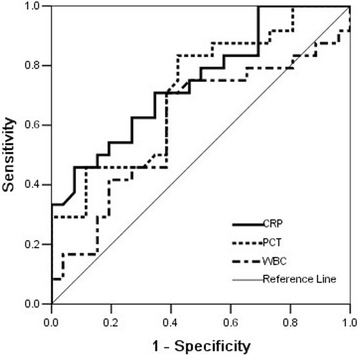
Diagnostic values for septic shock of hs-CRP, PCT, WBC, estimated by receiver operating curve (ROC) analysis. hs-CRP area 0.751 (95% CI 0.62-0.88), *P* = 0.002; PCT area 0.719 (95% CI 0.57-0.86), *P* = 0.007; WBC area 0.606 (95% CI 0.45-0.77), *P* = 0.165

No difference in the WBC was found between the two groups. The CRP level and PCT level in the septic shock group was significantly higher than that in the sepsis group. (Table 3) To evaluate the sensitivity and specificity of these three biomarkers, ROC curves were calculated (Fig. 2). The ROC analysis showed that hs-CRP was a good marker that discriminated septic shock patients from sepsis patients, with an AUC of 0.751 (95% CI: 0.62-0.88; *P* = 0.002). PCT was found to have an AUC of 0.719 (95% CI: 0.57-0.86; *p* = 0.007), which was not statistically different from CRP (z = 0.323, *p* = 0.747). The WBC was found to have an AUC of 0.613 (95% CI: 0.45-0.77; *p* = 0.165).

**Table 3:** Biomarkers between sepsis and septic shock group

Categories	Sepsis group(*n* = 27)	Septic shock group(*n* = 23)	*p* value
WBC (×10^9^/L)	11.5 (7.5-15.8)	12.5 (8.6-16.1)	0.282
CRP (mg/L)	118.1 (46.2-177.1)	180.2 (115.7-248.0)	0.003
PCT (ng/ml)	0.61 (0.27-2.07)	1.00 (0.71-25.03)	0.009

*WBC* white blood cell, *hs-CRP* high-sensitivity C-reactive protein, *PCT* procalcitonin

For serum hs-CRP, the optimum cutoff value for the diagnosis of septic shock was 139.4 mg/L, which resulted in a sensitivity of 70.8%, a specificity of 65.4%, PPV of 63.6% and an NPV of 72.4%. For serum PCT, the optimum cut-off value was 0.75 ng/ml, which resulted in a sensitivity of 70.8%, a specificity of 61.5%, a PPV of 61.4% and an NPV of 71.2%.

## Discussion

In this study, we found that hs-CRP was as good as PCT for the diagnosis of sepsis and septic shock among the oldest old patients. To our knowledge this is the first study that enrolled exclusively oldest old patients to evaluate and compare the diagnostic accuracy of PCT and CRP.

In the guideline issued by the Surviving Sepsis Campaign, sepsis and septic shock were labelled as medical emergencies, which necessitate immediate treatment and resuscitation [10]. The geriatric population is rapidly expanding, with the oldest old segment (≥85 yr) growing fastest [11]. Compared to adult patients, oldest old patients have fewer signs and symptoms of infection and more easily to develop organ failure [12]. Therefore, early diagnosis would be more meaningful in this population.

We noted in our study that leukocytosis was not a sensitive marker for sepsis or septic shock because no difference was observed between the non-sepsis and sepsis/SS groups. Febrile responses to infectious diseases in geriatric patients are often blunted or absent; the presence of fever may often indicate a significant infection, but the absence of fever does not reliably exclude serious illness [13]. Our study found that peak temperature was higher in the sepsis/SS group than in the nonsepsis group. However, no difference was found in the peak temperatures between the sepsis and septic shock groups.

The advantage of the PCT lies in its high specificity, with minimal or no increase in viral infection, cardiogenic shock, and non-infectious SIRS [14–16]. The C-reactive protein is one of the most widely used indicators for the response of acute-phase proteins. The measurement of serum CRP can help to differentiate inflammatory conditions from non-inflammatory conditions and are useful in managing the patient’s disease because the concentration often reflects the response to, and need for, therapeutic intervention [17]. The PCT and CRP levels were not well correlated in a prior study [18]. Another previous study found that PCT was better than CRP in a group of adult patients, with AUCs of 0.925 vs 0.677 for the diagnosis of sepsis. They discovered that SOFA scores were associated with PCT levels, *r* = 0.73, while the association with CRP was much lower, *r* = 0.41 [8]. Another study on critically ill neonates and children drew the same conclusion [19]. However, Steichen O et al. demonstrated that the clinical usefulness of PCT to diagnose invasive bacterial infections in elderly patients appears to be very limited [20]. Nouvenne A et al. noted that, for elderly patients, hs-CRP is more useful than PCT in diagnosing pneumonia [21].

In this study, we found that PCT was not better than hs-CRP in oldest old patients. Usually, PCT often has a high negative predictive value [22]. However, in this specific population, the negative predictive value of PCT was lower than hs-CRP both in the diagnosis of sepsis and in septic shock. A PCT cutoff of 0.5 ng/mL is frequently cited for the diagnosis of bacterial sepsis and bacteremia in adult patients [23, 24]. However, the PCT tends to be lower in elderly patients. In a group of elderly people (>65 yr), a PCT level of 0.2 ng/ml is sensitive for bacteraemia [25]. In this study, we also discovered that the best cut-off value for sepsis/SS was 0.45 ng/ml. Despite that CRP was deemed to be nonspecific, a previous study indicated that when there is a CRP level greater than 100 mg/L, 80-85% of patients have bacterial infections [26]. Additionally, CRP has a broad abnormal range, and we demonstrated that levels at or above 74.2 mg/L should indicate the suspicion of sepsis, while levels at or above 139.4 mg/L highly suggestive of septic shock in oldest old patients.

The definition of sepsis was adjusted by replacing systemic inflammatory response syndrome (SIRS) criteria with the sequential organ failure assessment (SOFA) score [27]. In this study, we found that CRP was well correlated to the SOFA score, which made hs-CRP an adequate biomarker to reflect the severity of sepsis in oldest old patients. The hs-CRP has often been deemed to be a nonspecific indicator of systemic inflammation and will be elevated in many non-infectious circumstances [28, 29]. Povoa P, et al. demonstrated in their study that plasma CRP levels were significantly related to the infectious status, and they noted the superiority of hs-CRP to BT and WBC in their study. They also found that a plasma CRP level of 50 mg/L or more was highly suggestive of sepsis in a group of adult patients with an average age of 61.3 years [30].

There are some limitations that should be considered. First, serial measurements of PCT and CRP were not performed. A serial blood test of CRP in previous study demonstrated the peak level of CRP was within 24-48 h during sepsis [31]. However, elevated levels of hs-CRP were detected within 12 h of ICU admission in this study. Furthermore, we also discovered a broad distribution of hs-CRP levels, which correlated well with SOFA scores. Several prior studies have also evaluated the same time point, and they also detected elevated CRP levels [19, 32, 33]. Second, the sample was not large enough because the oldest old patients are still a small proportion of ICU patients. Third, the sex was not equally distributed in this oldest old group because male patients accounted for the majority. More female patients need to be studied before the same conclusion can be used in this population.

## Conclusions

The results of the present study suggest that hs-CRP is not inferior to PCT in the diagnosis of sepsis and septic shock in oldest old patients.

## Abbreviations

AUC: Area under the curve
hs-CRP: High-sensitivity C-reactive protein
ICU: Intensive care unit
NPV: Negative predictive value
PCT: Procalcitonin
PPV: Positive predictive value
ROC: Receiver operating characteristic
SIRS: Systemic inflammatory response syndrome
SOFA: Sequential organ failure assessment
WBC: White blood cell
